# High-Throughput Platform for Detection of Neutralizing Antibodies Using Flavivirus Reporter Replicon Particles

**DOI:** 10.3390/v14020346

**Published:** 2022-02-08

**Authors:** Arlen-Celina Lücke, Anja vom Hemdt, Janett Wieseler, Carlo Fischer, Marie Feldmann, Simon Rothenfusser, Jan Felix Drexler, Beate Mareike Kümmerer

**Affiliations:** 1 Institute of Virology, Medical Faculty, University of Bonn, 53127 Bonn, Germany; arlen.luecke@uni-bonn.de (A.-C.L.); anja.vom_hemdt@ukbonn.de (A.v.H.); janett.wieseler@ukbonn.de (J.W.); marie.feldmann@yahoo.com (M.F.); 2 Institute of Virology, Charité-Universitätsmedizin Berlin, Corporate Member of Freie Universtät Berlin and Humboldt-Universität zu Berlin, 10117 Berlin, Germany; carlo.fischer@charite.de (C.F.); felix.drexler@charite.de (J.F.D.); 3 Division of Clinical Pharmacology, University Hospital, LMU Munich, 80337 Munich, Germany; simon.rothenfusser@med.uni-muenchen.de; 4 Unit Clinical Pharmacology (EKliP), Helmholtz Center for Environmental Health, 80337 Munich, Germany; 5 Martinovsky Institute of Medical Parasitology, Tropical and Vector-Borne Diseases, Sechenov University, 119435 Moskow, Russia; 6 German Center for Infection Research (DZIF), Associated Partner Site Berlin, 10117 Berlin, Germany; 7 German Center for Infection Research (DZIF), Associated Partner Site Bonn-Cologne, 53127 Bonn, Germany

**Keywords:** flavivirus, virus replicon particles, neutralization assay, high-throughput, diagnostics

## Abstract

Flavivirus outbreaks require fast and reliable diagnostics that can be easily adapted to newly emerging and re-emerging flaviviruses. Due to the serological cross-reactivity among flavivirus antibodies, neutralization tests (NT) are considered the gold standard for sero-diagnostics. Here, we first established wild-type single-round infectious virus replicon particles (VRPs) by packaging a yellow fever virus (YFV) replicon expressing Gaussia luciferase (Gluc) with YFV structural proteins in trans using a double subgenomic Sindbis virus (SINV) replicon. The latter expressed the YFV envelope proteins prME via the first SINV subgenomic promoter and the capsid protein via a second subgenomic SINV promoter. VRPs were produced upon co-electroporation of replicon and packaging RNA. Introduction of single restriction enzyme sites in the packaging construct flanking the prME sequence easily allowed to exchange the prME moiety resulting in chimeric VRPs that have the surface proteins of other flaviviruses including dengue virus 1–-4, Zika virus, West Nile virus, and tick-borne encephalitis virus. Besides comparing the YF-VRP based NT assay to a YF reporter virus NT assay, we analyzed the neutralization efficiencies of different human anti-flavivirus sera or a monoclonal antibody against all established VRPs. The assays were performed in a 96-well high-throughput format setting with Gluc as readout in comparison to classical plaque reduction NTs indicating that the VRP-based NT assays are suitable for high-throughput analyses of neutralizing flavivirus antibodies.

## 1. Introduction

Flaviviruses, the majority of which are arthropod-borne, represent an ongoing threat to public health [1]. Some of the well-known flaviviruses include yellow fever virus (YFV), dengue virus (DENV), Zika virus (ZIKV), West Nile virus (WNV), and tick-borne encephalitis virus (TBEV), which cause widespread morbidity and mortality. Despite an available vaccine against yellow fever (YF) [2], several outbreaks have been described over the last years. Reoccurring YF cases involving sylvatic cycles have been described in Brazil since 2016 [3,4,5,6]. Furthermore, outbreaks based on urban cycles were reported in Sub-Saharan African countries in 2019 and 2020 [7,8,9]. While YF is absent in the Asia-Pacific region, Dengue occurs in the Asia-Pacific region as well as Africa, the Americas, and the Eastern Mediterranean with Asia representing about 70% of the global burden of disease [10,11,12]. ZIKV was first discovered in 1947 [13] and has reemerged to public attention in 2015/2016 due to a large epidemic in Latin America where the infection-related occurrence of congenital microcephaly and Guillain-Barré syndrome were described [14,15]. While YFV, DENV, and ZIKV are mainly transmitted by *Aedes* spp. therefore primarily occurring in tropical and sub-tropical regions of the world, WNV is transmitted by *Culex* spp. which are distributed almost worldwide and are thus also found in temperate regions [1,16]. As a result, WNV has caused large epidemics in the United States, with the first one observed in New York in 1999 from where the virus spread further [17,18]. Within the last decade, a genetic WNV lineage 2 strain was spreading throughout Europe [19]. The virus was first detected in Central Europe and the Mediterranean [20]. In 2018, this WNV strain was also reported in Germany, mainly in different birds [21]. Another flavivirus for which numbers are increasing in Europe is TBEV, which is transmitted by ticks and is also endemic in large parts of Asia [22,23].

Flaviviruses are enveloped, positive-strand RNA viruses belonging to the family *Flaviviridae*. Their genome encodes one open reading frame which is flanked by 5′ and 3′ untranslated regions and translates into one polyprotein that is cleaved co- and post-translationally by host cell and viral proteases [24]. While the N-terminal part of the genome encompasses the structural proteins (C-prM-E), the remaining two C-terminal parts encode the nonstructural (NS) proteins (NS1-NS2A-NS2B-NS3-NS4A-2K-NS4B-NS5). Most of the cleavages in the structural protein region are mediated by the host cell signalase. Exceptions include cleavage of prM into pr and M by the host cell enzyme furin and cleavage generating the C terminus of the viral C protein by the viral serine protease (NS2B-3). This protease also mediates the majority of cleavages within the nonstructural protein region resulting in the production of the N-termini of NS2B, NS3, NS4A, 2K and NS5. The enzyme cleaving at the NS1/2A site is unknown.

Recurrent flavivirus outbreaks highlight the importance of reliable diagnostics. Several serological tests for either flavivirus antigen or antibody detection have been described, with the sandwich or indirect enzyme-linked immunosorbent assay probably being the most widely used [25]. However, a well-known problem is the observed cross-reactivity among flavivirus antibodies which represents a problem especially in hyperendemic settings [25]. Hence, for the differential serodiagnosis, the considered gold standard of flavivirus neutralization tests (NT) need to be performed. The classical NT assay is the plaque reduction neutralization test (PRNT). This assay involves the use of infectious virus and is time-consuming and laborious [26]. Alternatively, reporter viruses can be used that may allow less time-intensive NT assay procedures. However, several mosquito-borne flaviviruses are classified as Biosafety Level (BSL) 3 agents making a laboratory of the appropriate safety level necessary when working with infectious virus, irrespective of reporter genes. To circumvent the need for a BSL3 laboratory, NT assays based on single-round infectious virus replicon particles (VRPs) represent a useful alternative. VRPs are produced by packaging of subgenomic replicons lacking the structural protein region with structural proteins expressed in trans. Several VRPs have been described for flaviviruses, some of which have also been used in NT assays [27,28,29].

Here, we established VRP-based NT assays that allow analyses of neutralizing flavivirus antibodies in a high-throughput format for (re-)emerging flaviviruses.

## 2. Materials and Methods

### 2.1. Cells and Stable Cell Line

Baby hamster kidney cells (BHK-21/J cells, kindly provided by Charles M. Rice, NY, USA), were cultured in Minimum Essential Media (MEM, Gibco, Thermo Fisher Scientific, Waltham, MA, USA) supplemented with 7.5% fetal bovine serum (FBS, Gibco), 1% L-glutmine (Gibco), and 1% nonessential amino acids (Gibco), hereinafter named as MEM complete. Vero CCL-81 cells (ATCC, Manassas, VA, USA) and Vero B4 cells (kindly provided by Ute Winke, Bonn, Germany) were cultured in Dulbecco’s Modified Eagle’s medium (DMEM, Gibco) containing 1% L-glutamine and 10% FBS. Cells were maintained at 5% CO_2_ and 37 °C.

For the generation of a stable cell line expressing YFV prME and C, BHK-J cells were electroporated with in vitro transcribed RNA derived from the Sindbis virus (SINV) packaging construct SIN-YFprME/C plasmid (see below). To select for SINV replicon containing cells expressing YFV prME and C, the medium was replaced at 24 h post electroporation with MEM complete medium containing puromycin (AppliChem, Darmstadt, Germany) at 5 µg/mL. After selection, the cell population was passaged and maintained in MEM complete medium containing 5 µg/mL puromycin at 5% CO_2_ and 37 °C.

### 2.2. Viruses

For the PRNT assays, DENV serotypes 1–4 (kindly provided by Jonas Schmidt-Chanasit, Hamburg, Germany) as described previously [30], ZIKV MR-766 (kindly provided by Jonas Schmidt-Chanasit), WNV New York 1999 (kindly provided by Matthias Niedrig, Berlin, Germany), TBEV strain K23 (kindly provided by Petra Emmerich, Hamburg, Germany) and YFV-17D derived from pACNR/FLYF [31] were used. The YFV-Venus reporter virus was described in [32].

### 2.3. Plasmid Constructions

To generate a YFV replicon expressing Gaussia luciferase (Gluc), three PCR fragments were generated. The first fragment encompassing the 5′ terminal part of the YFV genome was amplified from a YFV Renilla replicon (YFVR) [33] using primers Bo286 and Bo1159 (see Supplementary Materials Table S1 for primer sequences). The second PCR amplified the Gluc gene from a CHIKV-Gluc replicon [34] using primers Bo1158 and Bo1161, and the third fragment was amplified from the YFVR replicon using primers Bo1160 and Bo88. The resulting three fragments were fused by an overlap extension PCR using the outer primers Bo286 and Bo88. The obtained PCR fragment was cut with NotI and MluI and cloned into YFVR cut with the same restriction enzymes.

To establish a packaging construct expressing YFV prME and C based on a double subgenomic noncytopathic SINV replicon [35], the YFV capsid region was amplified from pACNR/FLYF [31] using primers Bo905 and Bo907. In addition, the SINV 3′ UTR region was amplified from pSR19NS1-2A [36] using primers Bo906 and Bo908. After fusing the two PCR fragments using primers Bo905 and Bo908, the resulting fragment was cut with XbaI and ApaI and cloned together with a pSR19NS1-2A derived MluI-XbaI fragment into pSR19NS1-2A cut with ApaI and MluI resulting in pSR19YFV-C. Next, a codon-optimized puromycin N-acetyl-transferase (PAC) gene fused to a Thosea asigna virus 2A (T2A) sequence was amplified using Bo909 and Bo910. This fragment and fused together with a T2A-YFV capsid anchor (Ca)-prME encoding PCR fragment amplified from pACNR/FLYF with Bo911 and Bo912 using the outer primer pair Bo909 and Bo912. The resulting PuroR-T2A-YFV-prME cassette was cut with XbaI and MluI and cloned together with a Mlu-XhoI derived fragment from pSR19YFV-C into pSR19YFV-C cut with XhoI and XbaI resulting in pSR19/PuroR-T2A-YFVprME/C, which hereinafter is named SIN-YFprME/C.

To establish further SINV replicon-based packaging constructs that allow the easy exchange of the prME sequence, an SbfI restriction enzyme site was inserted after the T2A sequence resulting in an in frame Gly-Ala insertion on the protein level. For DENV-2 and ZIKV, Ca-prM-E cassettes were amplified from pD2/IC-30P-A [37] using primers CF1_D2f and CF2-D2r or pCCI-SP6-ZIKV [38] using primers Bo1555 and Bo1556, respectively. Viral RNA derived from DENV-1, DENV-3, and DENV-4 [30] served as templates for amplification of the respective Ca-prM-E cassettes via RT-PCR using primers Bo1061and Bo1031 (DENV-1), Bo1051 and Bo1052 (DENV-3), or Bo1047 and Bo1048 (DENV-4), respectively. Correspondingly, the Ca-prM-E cassette from WNV New York was amplified using primers Bo1557 and Bo1558, from ZIKV-MR766 using primers Bo1571 and Bo1562, and from TBEV strain K23 using primers Bo1566 and Bo1564. To obtain the different final packaging constructs, the amplified Ca-prM-E fragments were cut with SbfI and MluI and inserted into SIN-YFprME/C cut with the same restriction enzymes.

### 2.4. In Vitro Transcription and Electroporation

Linearization of the YFV-Gluc replicon and the SIN-flavi-prME/C packaging constructs was performed with XhoI. Linearized plasmids were transcribed in vitro using the SP6 mMessage Machine Kit (Invitrogen^TM^, Thermo Fisher Scientific) according to the manufacturer’s instructions. The integrity and amount of the in vitro transcribed RNA were assessed by electrophoresis in ethidium bromide agarose gels. Electroporation of in vitro transcribed RNA was performed as described previously [39].

### 2.5. Preparation and Titration of Flavivirus Reporter Replicon Particles

VRPs were produced by co-electroporation of in vitro transcribed YFV-Gluc replicon RNA and in vitro transcribed SIN-flavi-prME/C packaging construct RNA into BHK-J cells using the electroporation conditions as described [39]. After electroporation, cells were kept at 32 °C in MEM complete for 72 h. The VRPs were harvested by centrifugation of the cell supernatant at 1200 rpm for 10 min to remove cell debris. The cleared supernatants containing the VRPs were aliquoted and stored at −80 °C.

For the determination of VRP titers TCID_50_ analysis was performed. For this, the VRPs were serially diluted in tenfold steps in MEM with four replicates per dilution. In 96-well plates, an equal amount of BHK-J cell suspension (2 × 10^4^ cells/well) was added to each dilution and incubated at 37 °C, 5% CO_2_ for 48 h. Readout was performed via measurement of the Gluc activity using the *Renilla* Luciferase Assay System (Promega, Madison, WI, USA) and a plate luminometer (BioTek Synergy 2, Agilent, CA, USA). Wells with luciferase activity at least two-fold above the threshold measured for mock-infected cells were counted as positive. TCID_50_ was calculated by the Spearman and Kärber algorithm as described [40] and converted to focus forming units (FFU)/mL with FFU approx. 0.69 × TCID_50_.

### 2.6. Indirect Immunofluorescence

Cells were fixed in ice-cold methanol/acetone (1:1), dried and rehydrated in PBS. Anti-flavivirus group antigen antibody, clone D1-4G2-4-15, (Merck, Darmstadt, Germany) was diluted at 1:5000 in PBS and incubated at 4 °C overnight. Secondary fluorophore-labeled Alexa Fluor 555 goat anti-mouse IgG (H+L) (Life Technologies corporation, Carlsbad, CA, USA) was applied at 1:400 in PBS and incubated for 1 h at 37 °C. Nuclei were stained with 4′,6-diamidino-2-phenylindole (DAPI). Detection was performed using fluorescence microscopy (Zeiss Axio Imager M1, Jena, Germany).

### 2.7. Western Blot Analysis

Cells were washed with PBS and then lysed in Laemmli loading buffer (50 mM Tris-HCl, pH 6.8, 15% glycerol, 2% sodium dodecyl sulfate (SDS), 0.05% bromophenol blue, and 5 mM DTT), sheared through a 27-gauge needle (BD, Heidelberg, Germany) and heated for 10 min at 95 °C. The proteins were separated on a 10% SDS polyacrylamide gel and transferred to a nitrocellulose membrane (GE Healthcare, IL, USA). After blocking with 10% Roti block (Roth, Karlsruhe, Germany), incubation with the primary antibody rabbit anti-E2 YF 7/28/87 (kindly provided by C. M. Rice, Rockefeller University, New York, NY, USA) at 1:10,000 in PBS overnight at 4 °C followed. The secondary HPO-coupled goat anti-rabbit IgG antibody (Pierce, Thermo Fisher, Waltham, MA, USA) was applied at 1:10,000 in PBS for 1 h at room temperature. For detection, the SuperSignal™ West Femto Kit (ThermoFisher Scientific) was used according to the manufacturer’s protocol.

### 2.8. Neutralization Assays

For the VRP-based NT-assays, BHK-J cells were seeded at 2 × 10^4^ cells per well in 96-well plates the day before infection. VRP dilutions were adjusted to MOI 1, 3, or 5. Heat inactivated sera (30 min, 56 °C) were serially two-fold diluted in MEM complete and then mixed with an equal amount of VRP dilution, or directly serially two-fold diluted in the VRP solution (e.g., for VRP stocks with lower concentration). Serum dilution series started at 1:100 for the human anti-flavivirus sera (except anti-DENV-2—1:80) or at 1:400 for the anti-WNV mAb. After 1 h pre-incubation of VRPs with sera/mAb at 37 °C, the mixtures were added to the cell monolayers and incubated for another hour at 37 °C, 5% CO_2_. Following incubation, the inoculum was removed, cells were washed 1 x with PBS, and finally MEM complete was added. Supernatants were collected for Gluc analysis as described above at 24 h or 48 h post-infection as indicated. Neutralization of the sera or mAb was determined by the percental reduction of Gluc activity in sera/mAb samples compared to Gluc activity in untreated VRP samples.

To perform DENV and ZIKV PRNTs, VeroB4 (8 × 10^4^) cells were seeded the day before infection in 24-well plates. Heat inactivated serum was three-fold serially diluted starting at 1:50. Alternatively, WNV monoclonal antibody (mAb) was three-fold serially diluted starting at 1:200. The dilutions were mixed with the same volume of virus solution containing 60 plaque-forming units (PFU). After incubation for 1 h at 37 °C, the mixture was added to the cells and incubation was performed again for 1 h at 37 °C before cells were overlaid with a methylcellulose/MEM overlay (DENV: 2% FBS and 1.2% final methylcellulose; ZIKV: 2% FBS and 0.6% final methylcellulose). After incubation for five (DENV-2) or four (ZIKV) days, we performed formaldehyde fixation, crystal violet staining and plaque counting. PRNTs for YFV, WNV, and TBEV were performed likewise using BHK-J cells seeded in 6-well plates (YFV, TBEV: 3 × 10^5^ cells/well, WNV: 4 × 10^5^ cells/well). For each well, 100 PFU of virus were used and cells were overlaid with 0.6% agarose in MEM containing 2% FBS. Fixation and crystal violet staining to allow plaque counting were performed after three (YFV, TBEV) or two (WNV) days, respectively. Neutralization of the sera or mAb was determined by the percental reduction of plaques compared to plaque formation on infected cells incubated without sera/mAb.

For neutralization assays, human anti-YFV sera from routinely YFV vaccinated individuals, human anti-ZIKV and anti-DENV sera from INSTAND e.V. Düsseldorf, Germany, and mouse anti-YFV as well as mouse control serum (kindly provided by Danillo Espósito, São Paulo, Brazil) were used. In addition, a monoclonal antibody (mAb) directed against WNV E protein (kindly provided by Petra Emmerich. Hamburg, Germany) was tested.

### 2.9. Statistics

Spearman rank-order correlation to evaluate the relationship between two assays as well as non-linear regression analyses using the built-in variable slope model to calculate NT_50_ titers were performed using Prism 8 (GraphPad Software, Inc., San Diego, CA, USA).

## 3. Results

### 3.1. Generation of a YFV Replicon Expressing Gaussia luciferase

**Figure 1:**
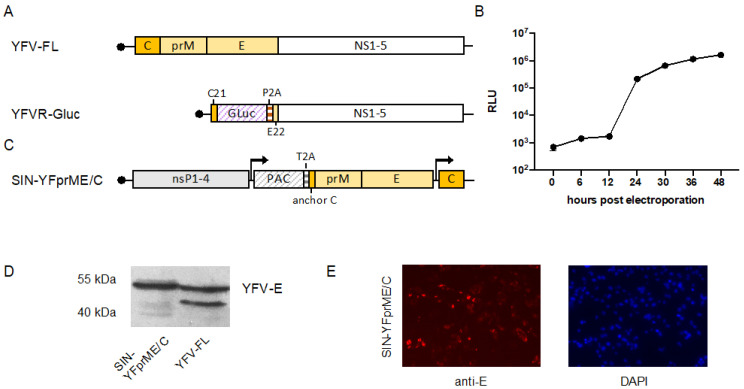
Scheme and characterization of components for yellow fever virus replicon particle (YF-VRP) production. (**A**) Schematic presentation of the YFV replicon expressing Gaussia luciferase (YFVR-Gluc) in comparison to the YFV full-length genome (YFV-FL). Lines indicate non-translated regions with the solid black dots at each 5′ end representing the cap structure. Boxes represent translated regions, with solid-colored boxes indicating the YFV structural proteins, the open box the YFV nonstructural proteins, and hatched boxes the Gluc marker or porcine teschovirus-1 derived autocatalytic peptide 2A (P2A), respectively. C21 represents the first 21 amino acids of the capsid protein and E22 the last 22 amino acids of the envelope protein, which serve as signal sequence for the following NS1 protein. (**B**) Kinetics of Gluc secretion after electroporation of the YFV replicon expressing Gluc. RNA transcribed in vitro from the replicon was electroporated into BHK cells and Gluc secreted into the supernatant was measured at the indicated time points (RLU: relative light units). (**C**) Scheme of the double subgenomic noncytopathogenic SINV replicon-based packaging construct expressing the YFV structural proteins (SIN-YFprME/C). Hatched boxes indicate the puromycin N-acetyl-transferase (PAC) or the Thosea asigna virus 2A self-cleavage peptide (T2A), respectively. Other symbols used as described under A. Arrows indicate the positions of the SINV subgenomic promoters. (**D**) Western blot analyses of YFV E in packaging cell line stably expressing YFprME/C. A lysate of cells infected with YFV served as a positive control. Detection was performed with a rabbit anti-YFV-E antiserum (**E**) Immunofluorescence analysis of the stable packaging cell line. Indirect immunofluorescence was performed using a pan-flavi antibody recognizing the envelope protein. Nuclei were stained using DAPI.

To establish a YFV reporter replicon allowing easy detection of replication, the majority of the YFV structural protein gene region was replaced by a cassette encoding a Gluc reporter. We chose a humanized Gluc because it was described to yield up to 1000-fold higher bioluminescent signal intensity compared to humanized Renilla luc or Firefly luc and is also secreted into the supernatant simplifying the readout [41]. The first 21 codons of the capsid gene as well as the last 22 codons of the envelope (E) gene were retained to allow efficient translation initiation or correct insertion of NS1 in the endoplasmatic reticulum (ER) lumen, respectively [42,43]. To mediate cleavage of the Gluc reporter from the polyprotein, a porcine teschovirus-1 derived 2A (P2A) autoprotease sequence was inserted 3′-terminally of the Gluc gene (Figure 1A). The functionality of the replicon was verified by electroporating in vitro transcribed RNA into BHK cells. Gluc in the supernatant was measured at several time points after electroporation yielding increasing luciferase levels over time, thereby confirming efficient replication of the replicon (Figure 1B).

### 3.2. Establishment of a Double Subgenomic YFV Packaging Construct

For the production of functional VRPs, the YFV structural proteins need to be provided in trans by a so-called packaging construct. Since the YFV replicon still contains the first 21 codons of the C protein (C21) as well as the last 22 codons of the E protein (E22), potential RNA recombination at these sequence elements could result in the emergence of infectious YFV. To prevent this, we established a double subgenomic alphavirus packaging construct based on a noncytopathogenic SINV replicon in which the order of the YFV structural proteins has been reversed. The first subgenomic mRNA encodes the envelope proteins prM and E and the second subgenomic mRNA encodes the capsid protein (Figure 1C). Furthermore, to enable the establishment of a cell line stably expressing the YFV structural proteins, we introduced a PAC gene under the control of the first subgenomic promoter upstream of the prM-E genes as a selection marker. Cleavage between the PAC and the structural protein region was mediated by insertion of a T2A self-cleavage peptide. Following transfection and selection with puromycin, we determined the successful expression of the YFV envelope proteins via the SINV replicon by immunodetection using western blot and immunofluorescence analyses (Figure 1D,E). As no YFV capsid-specific antibody was available, protein expression driven by the second SINV subgenomic promoter was verified indirectly in further experiments via successful packaging of the YFV replicon and thus production of functional YFV VRPs (see below).

### 3.3. Optimization of Flavivirus VRP Production

**Figure 2:**
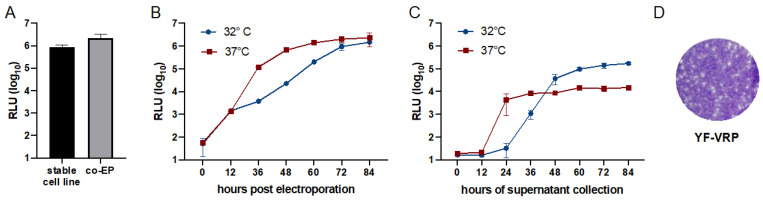
Optimization of VRP production. (**A**) In vitro synthesized YFV Gluc replicon RNA was either electroporated into BHK cells stably expressing the flavivirus structural proteins or co-electroporated with in vitro transcribed RNA derived from the packaging construct. Equal amounts of supernatant harvested 72 h after electroporation were used to infect naïve BHK cells. At 24 h after infection, Gluc activity released into the supernatant was measured. (**B**) In vitro transcribed YFV Gluc replicon RNA was co-electroporated with in vitro transcribed RNA from the packaging construct and cells were incubated at either 32 °C or 37 °C. At the indicated time points, supernatants were harvested and Gluc activity was measured. (**C**) Equal volumes of supernatants harvested at different time points from the different incubation temperatures in (**B**) were used to infect fresh BHK cells. At 24 h post-infection, Gluc activity was measured in the supernatant. (**D**) Plaque formation after titration of YF-VRPs on BHK cells stably expressing YFV structural proteins. After infection, cells were overlaid with agarose and incubated at 37 °C. After three days, cells were fixed and crystal violet staining was performed.

The PAC expressing double subgenomic packaging construct was primarily established to enable the production of a stable cell line expressing the YFV structural proteins to allow subsequent transfection of the YFV replicon. However, we considered whether co-electroporation of in vitro transcribed RNAs derived from the packaging construct (‘packaging RNA’) and the YFV replicon might result in a similar or even better VRP production. We therefore compared the VRP production after electroporation of the YFV replicon in the cell line stably expressing the YFV structural proteins to the VRP production obtained after co-electroporation. Using the VRPs released into the supernatants from both approaches for infection of fresh cells revealed that the co-electroporation approach yielded a 2.5-fold higher level of produced VRPs (Figure 2A) and therefore was used for further optimization of the VRP production. For alphaviruses, it has been observed that the production of VRPs is more efficient at temperatures lower than 37 °C [34]. Hence, for comparison, cells were either incubated at 37 °C or 32 °C after co-electroporation of the YFV replicon and packaging RNA. Measuring the Gluc activity released into the supernatant over time directly after electroporation revealed higher luciferase levels for incubation at 37 °C (Figure 2B). Passaging the supernatants harvested at different time points after electroporation on naïve BHK cells, however, revealed that at time points after 48 h higher amounts of infectious VRPs were produced at 32 °C compared to the experiment performed at 37 °C (Figure 2C). Using supernatant collected between 60 and 84 h for reinfection revealed that in this time window the VRP production reached a plateau (Figure 2C). Hence, for further VRP production, co-electroporated cells were incubated at 32 °C and VRPs were harvested at 72 h post electroporation. Functionality of the produced VRPs was also verified by titration on the cell line stably expressing YFV prM-E/C resulting in the formation of plaques (Figure 2D).

### 3.4. Set-Up and Validation of YF-VRP Based Neutralization Assays

**Figure 3:**
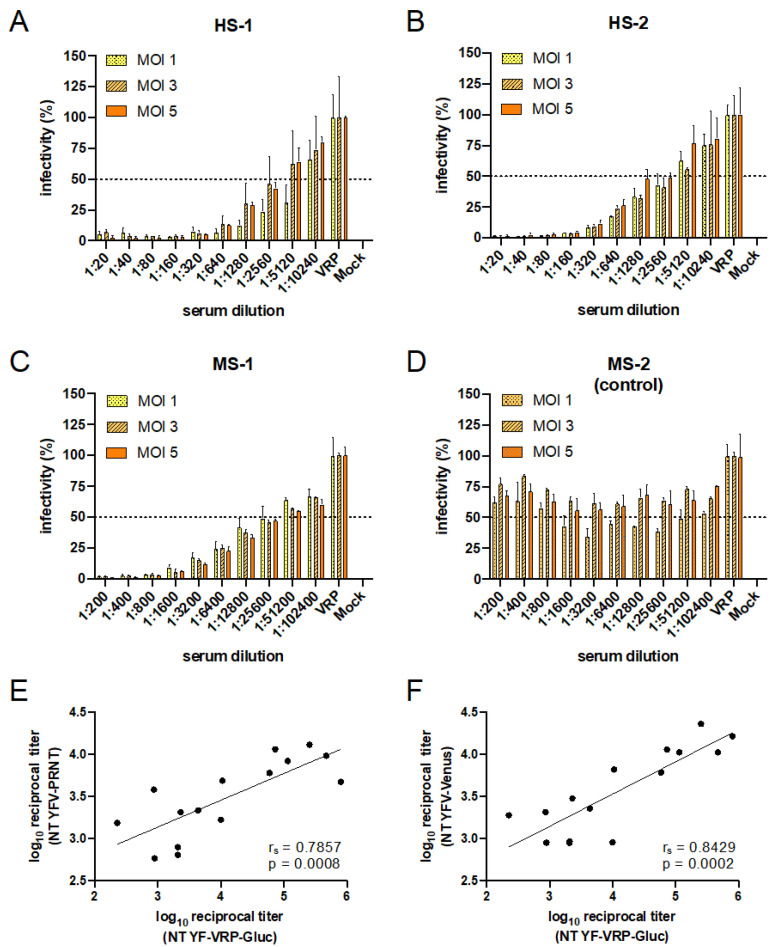
Validation of YF-VRP-Gluc based NT assay. (**A**,**B**) NT assays using sera from YFV vaccinated humans were performed in 96-well plates. Human sera were serially diluted and preincubated with VRPs corresponding to an MOI of 1, 3, or 5. BHK cells were infected with the preincubated samples and at 24 h post-infection readout via Gluc secreted into the supernatant was performed. For bars labeled with VRP, cells were infected with the appropriate amount of VRPs not preincubated with serum. The % infectivity was normalized to VRP infection without serum incubations. Data represent mean ± SD of experiments performed in duplicate. (**C**,**D**) YFV VRP-Gluc NT assays using an anti-YFV mouse serum (**C**) or a mouse control serum (**D**) were performed as described for (**A**,**B**). (**E**) Correlation of NT_50_ titers obtained via YF-VRP-Gluc NT assay (MOI 5) and NT_50_ titers obtained via PRNT assay. (**F**) Correlation of NT_50_ titers obtained via YF-VRP-Gluc NT assay (MOI 5) and NT_50_ titers obtained using a full-length YFV-17D-Venus reporter virus (MOI 1). (**E**,**F**) Depicted values are derived from duplicates. The lines indicate the linear regressions. r_s_: Spearman correlation coefficient.

Although VRP titration on the stable BHK SIN-YFprME/C cell line resulted in the formation of plaques, these were often small, diffuse and difficult to count. We therefore determined the VRP concentration of the produced VRP stocks by TCID_50_ titration using Gluc as readout. To set up and optimize a format suitable for VRP based NT assays, we tested two sera from humans vaccinated against YFV and one mouse anti-YFV serum using three different MOIs. Serum from a non-YFV infected mouse served as control (Figure 3D). In contrast to the non-neutralizing serum (Figure 3D), stepwise neutralization was obtained for all MOIs tested indicating the suitability of the VRP-Gluc based neutralization assay (Figure 3A–C).

To further validate the use of the established YF-VRPs in neutralization assays, we analyzed sera from YFV vaccinated humans using YF-VRPs at an MOI of 5 and compared the NT_50_ titers to the results obtained by a classical PRNT assay and by a fluorescence-based NT assay using a Venus-tagged YFV 17D reporter virus [32]. As shown in Figure 3E,F, Spearman rank-order correlation analyses revealed strong positive, statistically significant associations between the NT_50_ titers obtained using the VRP-based assay and the PRNT-based assay (r_s_ = 0.7857; *p* = 0.0008) or the Venus-tagged YFV 17D-based NT assay (r_s_ = 0.8429; *p* = 0.0002), respectively.

### 3.5. Establishment and Validation of Chimeric Flavivirus VRPs for Neutralization Assays

**Figure 4:**
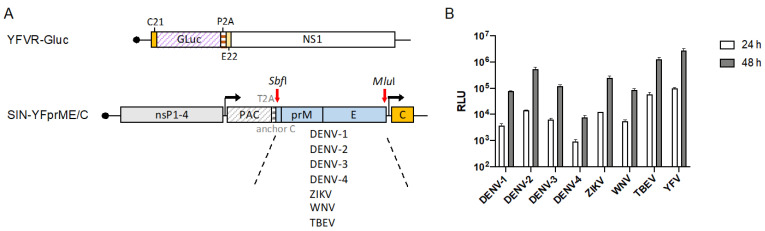
Production of chimeric VRPs. (**A**) Schematic presentation of co-electroporated RNAs used to produce chimeric VRPs, in which a YFV-Gluc replicon is encapsidated with envelope proteins of other flaviviruses. Different packaging RNAs based on a double subgenomic SINV replicon were used, each expressing the Ca-prM-E sequences of the indicated viruses and the YFV capsid protein. See also Figure 1 for further explanations. (**B**) Equal volumes of supernatant harvested after co-electroporation were used to infect fresh BHK cells. Gluc activity was measured in the supernatant at the indicated time points after infection. Data represent means ± SD of experiments performed in duplicate.

Besides YFV other flaviviruses such as DENV, ZIKV, WNV, and TBEV are a recurring threat in certain areas. To establish VRPs expressing the respective structural proteins necessary to perform virus-specific NT assays, we exchanged the YFV 17D Ca-prM-E cassette in our original packaging construct with the corresponding sequences of all four DENV serotypes (DENV-1-4), ZIKV, WNV, and TBEV. For easier exchange, an SbfI restriction enzyme site was introduced between the PAC gene and the Capsid anchor preceding the prM-E region (Figure 4A). Furthermore, the construct had an MluI restriction site at the 3′ end of the E gene, allowing the exchange of the SbfI-MluI fragment.

To verify the production of chimeric VRPs, YFVR-Gluc RNA was co-electroporated with each of the different packaging RNAs. Equal amounts of supernatants harvested three days post electroporation were used to infect naïve BHK cells and release of Gluc was measured at 24 and 48 h after infection (Figure 4B). Although the Gluc levels differed to a certain extent for the different VRPs, Gluc values increased in all cases between 24 and 48 h indicating the successful production of infectious chimeric VRPs. Titration of the chimeric VRPs yielded titers between 2.2 × 10^5^ to 3.9 × 10^6^ FFU/mL, while for our YF-VRPs titers up to 6.9 × 10^6^ FFU/mL were obtained.

**Figure 5:**
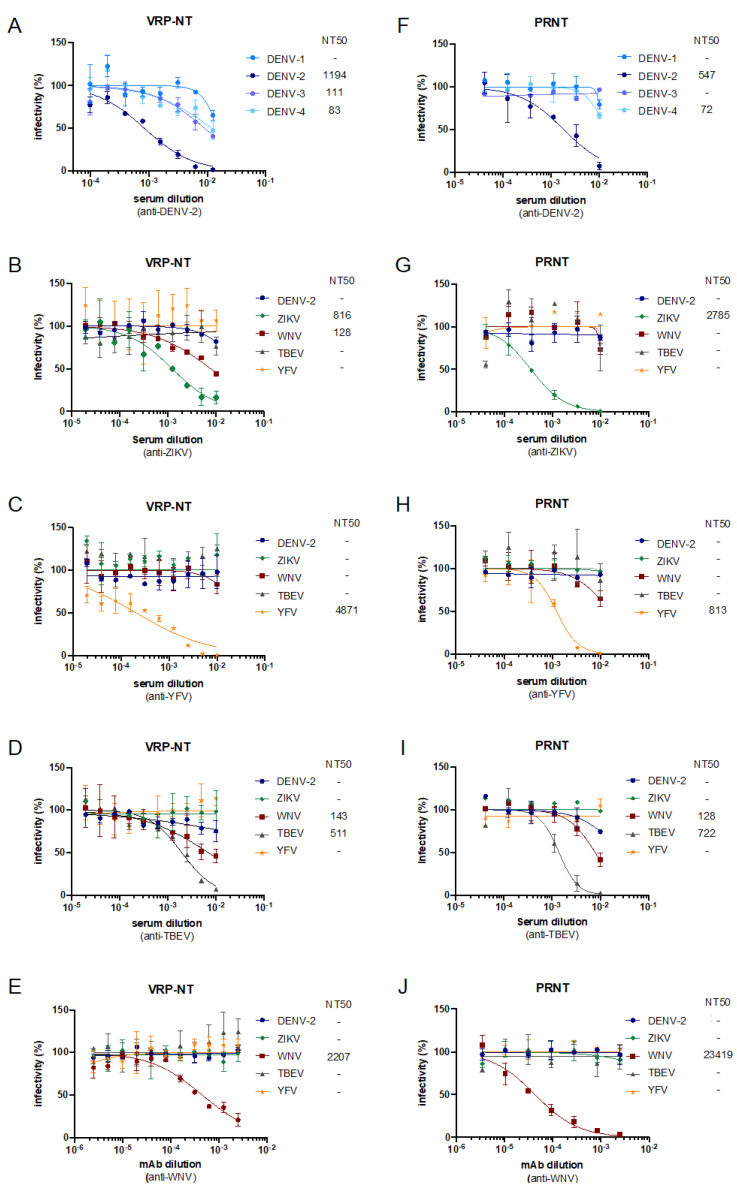
Comparison of VRP-based NT assays to plaque reduction NT assays. (**A**–**E**) Neutralization curves for VRP-based NT assays or (**F**–**J**) neutralization curves for PRNT-based NT assays using the indicated human anti-flavivirus sera or an anti-WNV mAb. VRP-based NT assays (MOI 1) were performed using two-fold serum dilutions and PRNT assays were done using three-fold serum dilutions. After preincubation of sera/mAb with VRPs/virus, BHK cells were infected with the preincubated samples. At 48 h post infection readout via Gluc secreted into the supernatant was performed. Normalized percentage neutralization values are plotted against the serum dilution factors. Inferred NT_50_ titers are depicted as the reciprocal of the serum dilution conferring 50% inhibition of VRP or virus infection. Data represent means ± SD of experiments performed in duplicate.

To validate the applicability of the VRPs, neutralization assays were performed using human sera against DENV-2, ZIKV, YFV, TBEV and a monoclonal antibody against WNV (Figure 5A–E). Since the titers of some chimeric VRP stocks were lower compared to the YF-VRP titers, the VRP assays were done using an MOI of 1. For comparison, PRNTs were performed using the corresponding wild-type viruses (Figure 5F–J).

Testing the anti-DENV-2 serum against all four DENV-VRPs revealed the highest neutralization efficiency against DENV-2 VRPs and some cross-reactivity to the other DENV serotypes (Figure 5A). In accordance, the anti-DENV-2 serum most efficiently also neutralized wild-type DENV-2 (Figure 5F). Similarly, testing DEN-, ZIK-, TBE-, WN-, and YF-VRPs with human sera against the respective different flaviviruses or the WNV mAb showed in each case the highest neutralization efficiency against the corresponding VRPs (Figure 5B–E) or the corresponding wild-type virus (Figure 5F–J), respectively. For the human sera, certain levels of cross-neutralization were observed for both the VRP-based NT assays as well as the PRNTs. Using the anti-WNV mAb only WN-VRPs or WNV were neutralized, respectively.

## 4. Discussion

In this study, we demonstrated that wild-type and chimeric flavivirus reporter replicon particles can be produced by packaging a YFV reporter replicon expressing Gluc with flavivirus structural proteins expressed by a double subgenomic SINV expression vector. The resulting VRPs are single-round infectious, which is of great value since several members of the family *Flaviviridae* of medical importance are classified as BSL3 agents. Particularly, this is important for flavivirus diagnostics, as cross-reactivity is a well-known problem and the NT assay has been described as the most specific assay among serological tests [25,26,44]. The classical NT assay is the PRNT, which is performed using infectious wild-type or mutant virus. Alternatively, genetically engineered flaviviruses have been described expressing a reporter protein e.g., a fluorescent or luminescent protein and neutralization is evaluated via reduction of the reporter protein signal [45]. Even if the reporter viruses simplify the readout, they are still classified into the same BSL category as the corresponding wild-type virus. In contrast, the single-round infectious VRPs allow to circumvent a BSL3 laboratory even if they are derived from a BSL3 virus since they do not result in the production of progeny virus [46].

Regarding VRP production, several types of constructs have been described that allow the production of flavivirus VRPs. This applies to both the replicons as well as the packaging constructs. Besides SP6 driven replicon constructs, CMV-promoter driven replicons have also been described [27,29,42,47]. The latter allows direct transfection of DNA but also carry the risk of splicing since transcription takes place in the nucleus. Transfecting RNA in vitro transcribed by SP6 or T7 RNA polymerase is more in line with the nature of RNA viruses, for which transcription usually takes place in the cytoplasm. Regarding the packaging systems, SP6- as well as CAG- or CMV-driven promoter constructs have been described, some of which only express prME since the capsid protein is still encoded by the replicon [27,42,47,48]. However, for most described flavivirus replicons the majority of the structural proteins are deleted so that it is necessary to express all structural proteins via a helper plasmid. This was either done by expressing CprME in one reading frame, by using two constructs expressing C and prME, respectively, or by using a bicistronic expression vector [27,29,47]. We chose the latter because on one hand it has been described that the yield of VRPs is tenfold higher when the structural proteins are expressed from one plasmid instead of two [27]. Furthermore, the latter construct allows to easily change the order of the expressed proteins in comparison to the original genome organization. This can be of importance to prevent recombination which could result in the production of infectious particles. For flaviviruses, this is relevant since the first 20 codons of the capsid protein containing the cyclization sequence as well as the last codons of the E protein functioning as signal sequence for NS1 and must be retained to allow replication [42,43]. Hence, expression of an in frame CprME cassette would yield overlapping sequences that could result in recombination and subsequently infectious particle production. In our setting, we used a double subgenomic SINV vector and cloned the prME genes under the control of a first 26S subgenomic promotor and the C gene under a second. The expressed capsid protein corresponds to the mature capsid lacking the anchor sequence. The latter is retained in the prME moiety to allow correct translocation and processing of the envelope proteins. Therefore, expressing the capsid protein individually bypasses the need for cleavage at the N-terminal anchor site, which is a prerequisite for proper cleavage at the prM signal sequence by the cellular signal peptidase [49]. In settings where CprME is expressed as one polyprotein, cleavage to remove the capsid anchor needs to be mediated in trans by the replicon expressed NS2B-3 protease.

Previous studies described a cell line inducible expressing flavivirus CprME [48]. In our double subgenomic packaging vector, a PAC gene is included under the control of the first subgenomic promotor. This allowed us to select a cell line stably expressing the flavivirus structural proteins. Titration of established YF-VRPs on this cell line resulted in production of plaques, which indicates the successful expression of the structural proteins and enables the determination of the VRP titer. However, the plaques were often small and indistinct, so we rather determined the VRP titer by endpoint titration and measurement of the Gluc activity. To produce VRP stocks, co-electroporation of the replicon RNA and the packaging construct derived RNA was chosen as it showed higher VRP titers compared to electroporation of the stable cell line. Besides packaging the YFV reporter replicon with YFV structural proteins, we also produced chimeric flavivirus replicons by encapsidating the YFV replicon with envelope proteins of other flaviviruses such as DENV, ZIKV, WNV, and TBEV. Similar approaches have been described before, in which either a JEV or DENV replicon was packaged by envelope proteins of other flaviviruses [28,46].

As mentioned, we obtained VRP titers between 10^5^ and 10^6^ FFU/mL, which were largely comparable to the VRP titers described by others ranging from 10^4^ to 10^9^ FFU/mL [27,28,48]. However, it should be noted that the titers are only comparable to a limited extent since different methods were used to evaluate the titration (e.g., immunofluorescence, luminescence, or ELISA). In any case, the obtained VRP titers were sufficiently high to perform neutralization assays using an MOI of 1. Testing human sera or a monoclonal antibody against different flavivirus VRPs showed in each case that the VRPs corresponding to the respective antiserum were most effectively inhibited as was also observed for the PRNTs. The NT_50_ titers, however, are not identical which is well known between different NT assay approaches. They can be affected even within one assay type such as the PRNTs by the plate format (24- or 6-well format) and the associated difference in the amount of virus used. Similarly, in a Chikungunya VRP-based NT assay, the NT_50_ values differed as well when using different plate formats (96- versus 24-well format) [34]. Still, the strength of neutralizing activities of the sera by means of NT_50_ titers correlated between the different assay formats. We also demonstrated this when comparing the NT_50_ values obtained via our YF-VRP based assay with the NT_50_ values obtained for the PRNT assay or YF-Venus reporter virus NT assay (Figure 3E,F).

We used Gluc as reporter protein instead of green fluorescent protein (GFP) or NanoLuc, which were mentioned for VRP-based NT assays earlier [29,46]. Besides the high sensitivity of the humanized Gluc and the advantage of secretion, it was also described that Gluc is very stable allowing storage of the supernatant at 4 °C for several days without significant loss of activity [41]. In addition, Gluc allowed an earlier readout of the NT assays compared to others. While our readout was performed at 48 h post-infection at the latest, others described readout times for VRP infection experiments of 3 or even 5–6 days using NanoLuc or GFP, respectively [29,46]. To readout the Gluc-based NT assays, a luminometer needs to be available which could be a limiting factor, especially in developing countries. In this case, an alternative option might be the use of a reporter protein which can be detected via color change such as secreted alkaline phosphatase (SEAP). A flavivirus replicon expressing SEAP has been described previously [50]. However, SEAP was found to be over 20.000-fold less sensitive compared to Gluc and the linear range covers fewer orders of magnitude than Gluc [51]. Therefore, analyses have to be performed to see whether the reporter protein SEAP is suitable for NT assays.

Altogether, using Gluc as reporter protein we were able to validate the use of our VRPs for NT assays in a high-throughput format using a limited number of sera or a monoclonal antibody against different flaviviruses. Our results were comparable to PRNT assays and the implementation does not require a BSL3 laboratory and is less time-consuming which makes the VRP-based NT assays a good alternative to PRNTs.

## 5. Conclusions

We have established a VRP-based NT assay against multiple flaviviruses by encapsidating a YFV replicon expressing Gluc with either the YFV envelope proteins or the envelope proteins of other flaviviruses such as DENV, ZIKV, WNV, or TBEV. The VRP-based NT assays can be done in a 96-well format using secreted Gluc as the readout making it suitable for high-throughput analyses of flavivirus antibodies.

## Data Availability

Not Applicable.

## Supplementary Materials

The following are available online at https://www.mdpi.com/article/10.3390/v14020346/s1, Table S1: List of primers used in this study.
