# Supplementary material for: High-Throughput Platform for Detection of Neutralizing Antibodies Using Flavivirus Reporter Replicon Particles
Source: Viruses. 2022 Feb 8;14(2):346. doi: 10.3390/v14020346 (PMC8880525; doi:10.3390/v14020346)
Supplement: Supplementary file 1 [file viruses-14-00346-s001.zip › viruses-1546559-supplementary.pdf]

Table S1: List of primers used in the study

Name	Sequence (5′-3′)
Bo88	TTGGAGAGCCATGGGCACTC
Bo286	GTTATTGTCTCATGAGCGGATAC
Bo905	TGCTCTAGAGCCGGCATGTCTGGTCGTAAAGCTCAGGGAAAAACCCTG
Bo907	GGCGCTGCGGGTCGTGGGGCGGGCGGCGGCCGCTCAACGGCGTTTCCTTGAGGACAAT CCT
Bo906	AGGATTGTCCTCAAGGAAACGCCGTTGAGCGGCCGCCGCCCGCCCCACGACCCGCAG CGCC
Bo908	CATTATGCACATCAGTTCCTCGGAAG
Bo909	GCTCTAGAGCCACCATGATGACCGAATACAAGCCCACGGTGC
Bo910	GGGCCCGGGATTTTCCTCCACGTCCCCGCATGTTAGAAGACTTCCCCTGCCCTCGGCTC TGGCACCGGGCTTGCGGGTCATGCAC
Bo911	AGAGCCGAGGGCAGGGGAAGTCTTCTAACATGCGGGGACGTGGAGGAAAATCCCGGG CCCTCCCATGATGTTCTGACTGTGCAAT
Bo912	ACTGTAACGCGTCTACGCCCCAACTCCTAGAGACAAAAACATC
Bo1031	ACAACGCGTCTATGCCTGAACCATGACTCCTAGGTACAGTG
Bo1047	TTACCTGCAGGATCAACAATGACATTGCTGTGCTTGATTCC
Bo1048	TAACGCGTTTATGCGTGAACTGTGAAACCCAGAAACAG
Bo1051	TTACCTGCAGGAACATCGCTCTGTCTCATGATGATGTTACC
Bo1052	TAACGCGTTTAAGCTTGCACCACGGCTCCCAGATAG
Bo1061	CCGGGCCTGCAGGATCCGTGACCATGCTCCTTATGCTGCTG
Bo1158	GCGTCAATATGGTACGACGAGGAGTACGTAACATGGGAGTCAAAGTTCTGTTTGCCCT GATCTGC
Bo1159	GCAGATCAGGGCAAACAGAACTTTGACTCCCATgttaCGtACTCCTCGTCGTACCATATT GACGC
Bo1160	AGATCAAGGGGGCCGGTGGTGACGGAAGCGGAGCTACTAACTTCAGCC
Bo1161	GGCTGAAGTTAGTAGCTCCGCTTCCGTCACCACCGGCCCCCTTGATCT
Bo1555	GAACCTGCAGGCGCAGATACTAGTGTCGGAATTG
Bo1556	TTCACGCGTCTAAGCAGAGACGGCTGTGGATAAGAAG
Bo1557	TGATCCTGCAGGAGGAAAGACCGGAATTGCAGTCA
Bo1558	TAGTACGCGTCTAAGCGTGCACGTTCACGGAGAGGAA
Bo1562	TAGTACGCGTCTAAGCAGAAACAGCCGTGGAGAGGAAG
Bo1564	TAGTACGCGTCTACGCCCCCACTCCAAGGGTCATGGCC
Bo1566	TGATCCTGCAGGATCAGCGACGGATTGGATGAGCTGGT
Bo1571	TGATCCTGCAGGACGTGGCGCAGACACCAGCATCGGAA
CF1_D2f	CAAGACCTGCAGGATCTGCAGGCATGATCATTATGCT
CF2-D2r	CATTAACGCGTCTAGGCCTGCACCATGACTCCCAAATAC
